# Multifocal Enterolithiasis Associated With Appendicitis and Colonic Diverticulosis Mimicking Gallstone Ileus: A Case Report

**DOI:** 10.7759/cureus.116948

**Published:** 2026-09-25

**Authors:** Mauro A Cardenas, Violeta G Beltran Nuñez, Roberto G Guzman Armendariz, Angel Portilla, Akemy H Tsuyi Loya, Katia Ramírez Martínez, Luis F Ochoa, Valeria Muñoz Salas, Jesus A Jacquez Graff, Luis D Chavarria Muñoz, Melisa Licon Meza, Luisa F Peña Martinez, Sergio Miguel Suárez Fuentes, Jazmin Sáenz López, Paola I Cárdenas Rosales, Luis A Muñoz Sanchez, Diana I Escalante Zamora, Bruno A Guggiari, Jose A Ojeda Valenzuela, Mario Hernandez Mancillas, Martín Rodríguez-Garza

**Affiliations:** 1 Surgery, Hospital General Presidente Lázaro Cárdenas del Rio Instituto de Seguridad y Servicios Sociales de los Trabajadores del Estado (ISSSTE), Chihuahua, MEX; 2 General Surgery, Autonomous University of Chihuahua, Chihuahua, MEX; 3 Surgery, Autonomous University of Chihuahua, Chihuahua, MEX; 4 Research, NeuroteamCUU (Chihuahua), Chihuahua, MEX; 5 Surgery, Hospital General Presidente Lázaro Cárdenas del Rio, Chihuahua, MEX; 6 Surgery, Institute for Social Security and Services for State Workers, Chihuahua, MEX; 7 General Surgery, Hospital Regional Valentín Gómez Farías, Zapopan, MEX; 8 General Medicine, Autonomous University of Chihuahua, Chihuahua, MEX; 9 Medicine, Autonomous University of Chihuahua, Chihuahua, MEX; 10 Internal Medicine, Hospital General Dr. Salvador Zubirán Anchondo, Chihuahua, MEX; 11 Pediatric Medicine, Hospital Infantil de Especialidades de Chihuahua, Chihuahua, MEX; 12 Thoracic Surgery, Hospital Civil de Guadalajara, Guadalajara, MEX; 13 Surgery, Hospital Civil de Culiacán, Culiacán, MEX; 14 General Surgery, Hospital General Instituto de Seguridad y Servicios Sociales de los Trabajadores del Estado (ISSSTE), Zacetecas, MEX; 15 General Surgery, Instituto de Seguridad y Servicios Sociales de los Trabajadores del Estado (ISSSTE), Monterrey, MEX

**Keywords:** appendicitis, cholelithiasis, diverticular disease, enterolithiasis, enteroliths, gallstone ileus

## Abstract

Enterolithiasis is an uncommon condition that can present with nonspecific symptoms and pose a diagnostic challenge when concomitant cholelithiasis suggests gallstone ileus. We report the case of a 58-year-old woman who presented with abdominal pain, vomiting, and obstipation, with computed tomography findings initially suggestive of gallstone ileus. Exploratory laparotomy revealed no small-bowel obstruction or obstructing calculus. Instead, multiple calculi were identified within the appendix, and additional calculi were found impacted within diverticula throughout the colon, consistent with multifocal enterolithiasis. Appendectomy was performed, and the diverticular calculi were reduced into the intestinal lumen. Cholecystectomy was deferred because the biliary anatomy could not be safely identified. Histopathological examination demonstrated acute fibrinopurulent appendicitis and periappendicitis despite the absence of evident macroscopic inflammation. This case illustrates an unusual multifocal distribution of enteroliths involving the appendix and multiple colonic diverticula in a patient with concomitant cholelithiasis. It underscores the importance of integrating radiological findings with systematic intraoperative exploration when differentiating enterolithiasis from gallstone ileus.

## Introduction

Enterolithiasis is an uncommon condition characterized by the formation of concretions within the gastrointestinal tract. Its development is primarily associated with conditions that promote intestinal stasis, such as diverticular disease, intestinal diverticula, surgical anastomoses, blind intestinal loops, and strictures. Clinically, enterolithiasis may remain asymptomatic or present with abdominal pain, nausea, vomiting, and, in complicated cases, intestinal obstruction, perforation, or bleeding [1].

Enteroliths can be classified as primary, when they form within the gastrointestinal tract as a consequence of stasis and anatomical abnormalities, or secondary, when they originate from other organs and migrate into the gastrointestinal tract, primarily through a fistulous communication [1]. Diagnosis may be challenging, particularly when concomitant cholelithiasis is present and computed tomography demonstrates hyperdense intraluminal structures [1,2].

Computed tomography plays an important role in identifying enteroliths and their potential complications. Although enterolithiasis is an uncommon cause of intestinal obstruction, radiological findings may raise suspicion for the diagnosis and help guide management [1,2].

A relevant diagnostic challenge arises when enterolithiasis coexists with cholelithiasis, as gallstone ileus may initially be suspected. Primary enteroliths have been reported to mimic gallstone ileus clinically and radiologically, particularly when no biliary-enteric fistula is identified [3]. This highlights the importance of correlating computed tomography findings with biliary anatomy and intraoperative findings [3,4].

We present a rare case of multifocal enterolithiasis involving the appendix and multiple colonic diverticula in a patient with concomitant cholelithiasis whose initial clinical and radiological presentation raised suspicion for gallstone ileus. Systematic intraoperative exploration revealed no obstructing calculus or mechanical transition point in the small bowel but identified multiple calculi within the appendix and five additional calculi within colonic diverticula. Histopathological examination subsequently demonstrated acute fibrinopurulent appendicitis and periappendicitis despite the absence of evident macroscopic inflammatory changes. This case highlights the diagnostic challenge posed by the coexistence of enterolithiasis and cholelithiasis and emphasizes the importance of integrating radiological and intraoperative findings.

## Case presentation

A 58-year-old woman with a medical history of systemic arterial hypertension, recently diagnosed hypercholesterolemia without pharmacological treatment, and a chronic sleep disorder treated with benzodiazepines presented for evaluation. Her surgical history included bilateral tubal ligation and hysterectomy, both performed several years earlier.

Her current illness began with colicky abdominal pain initially localized to the left iliac fossa, radiating to the ipsilateral lumbar region, with a reported intensity of 3/10 on the Numeric Rating Scale (NRS). Four days later, the intensity and frequency of the pain progressively increased, reaching 9/10 on the NRS and becoming associated with nausea and two episodes of gastric-content vomiting. During the 24 hours preceding admission, she experienced progressively decreased passage of flatus, eventually developing complete obstipation. Due to the progression of symptoms, approximately one week after symptom onset, she was initially evaluated by a primary care physician and subsequently referred to the emergency department for diagnostic evaluation and assessment by the general surgery service.

Upon admission, the patient was hemodynamically stable and had no clinical evidence of systemic inflammatory response. The abdomen was distended, with tenderness on moderate and deep palpation, predominantly involving the left hemiabdomen, without signs of peritoneal irritation. Bowel sounds were absent on auscultation. Laboratory studies showed no significant leukocytosis or other relevant biochemical abnormalities. The main laboratory findings are summarized in Table 1. Urinalysis showed bacteria, moderate crystals, and two red blood cells per high-power field, without clinical findings suggesting an acute urinary process as the primary cause of her symptoms.

**Table 1:** Laboratory findings on admission

Laboratory test	Patient’s value	Reference range	Units
Hemoglobin	15.6	14-18	g/dL
Platelets	230	150-400	×10³/µL
Leukocytes	6.42	4.5-10.5	×10³/µL
Neutrophils %	53	41.4-73	%
Creatinine	0.8	0.7-1.2	mg/dL

Computed tomography of the abdomen and pelvis was performed and initially lacked formal interpretation by a radiologist. Upon review of the images, three hyperdense intraluminal structures were identified within small-bowel loops in the right hemiabdomen, measuring approximately 7 × 7 mm, 5 × 5 mm, and 10 × 8 mm (Figure 1).

**Figure 1:**
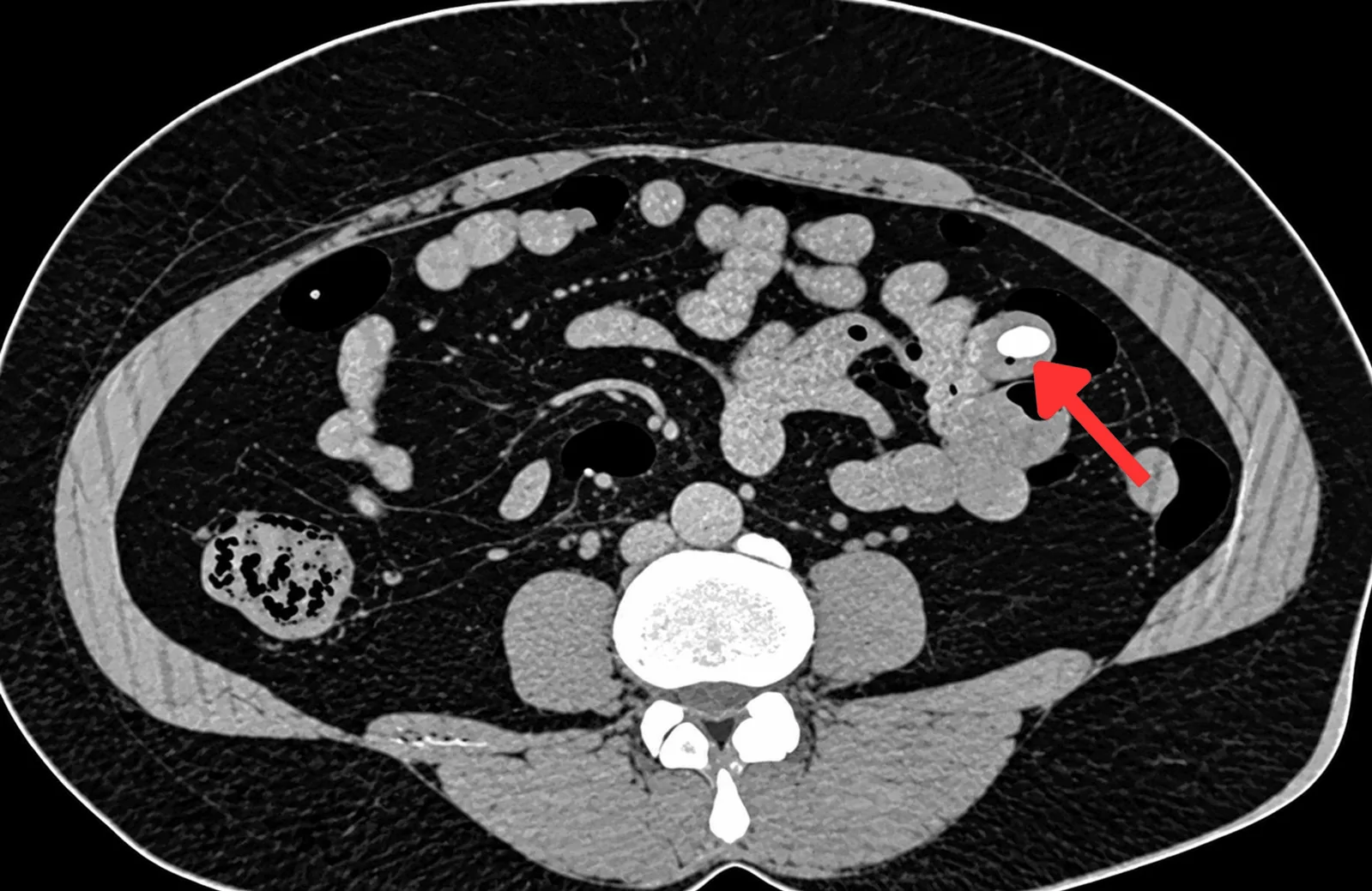
Admission abdominal computed tomography Computed tomography of the abdomen showing a hyperdense intraluminal structure within a small-bowel loop (arrow).

A hyperdense intraluminal structure was also identified within the gallbladder, with multiple additional hyperdense structures consistent with cholelithiasis, including a larger calculus apparently impacted at the gallbladder neck (Figure 2). No free intraperitoneal air or fluid was identified. Given the concomitant presence of hyperdense intraluminal structures within the small bowel and gallbladder cholelithiasis, a presumptive diagnosis of gallstone ileus was made, and exploratory laparotomy was performed.

**Figure 2:**
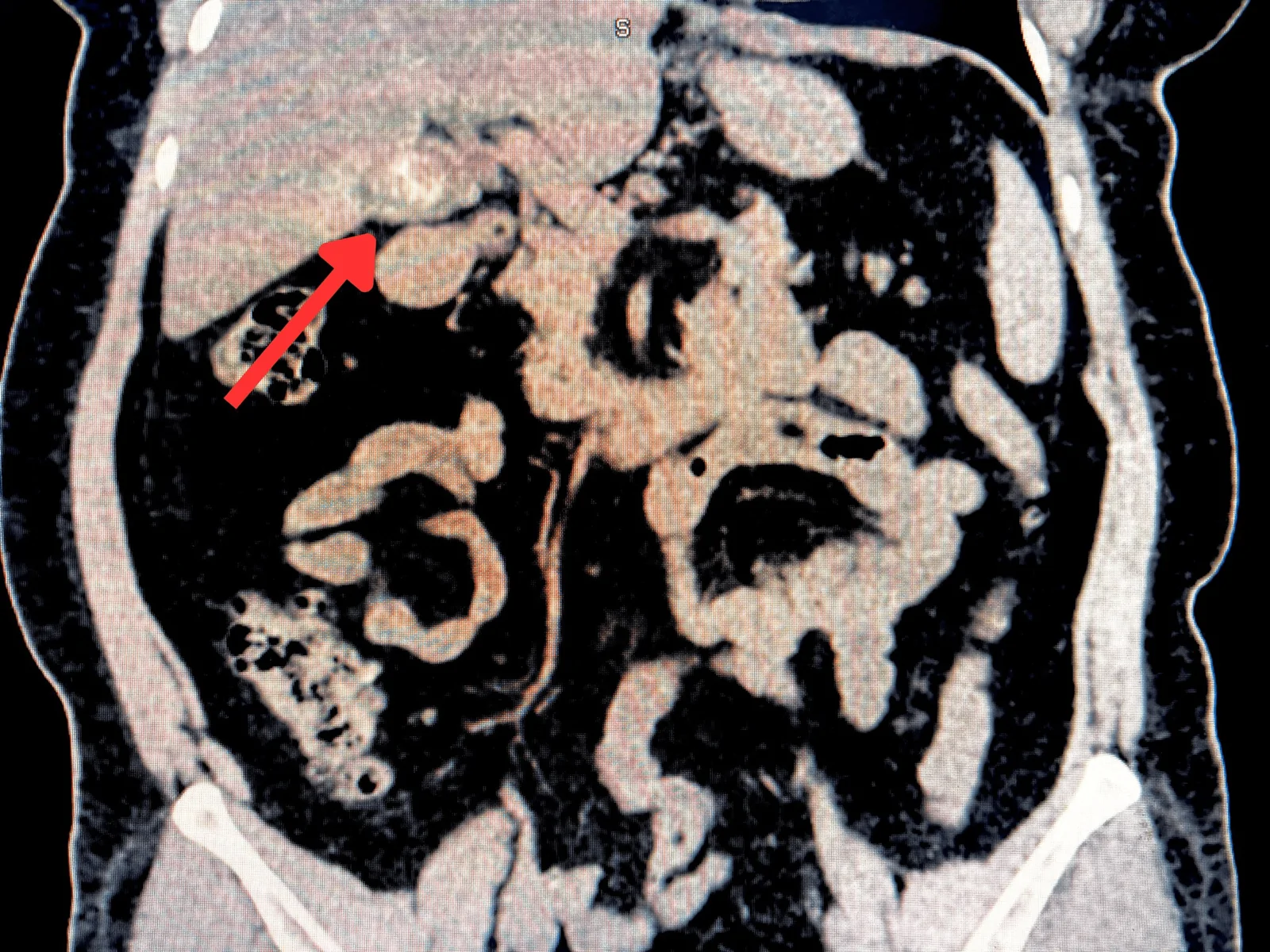
Coronal computed tomography demonstrating gallbladder cholelithiasis Coronal computed tomography image demonstrating multiple hyperdense calculi within the gallbladder, including a larger calculus apparently impacted at the gallbladder neck (red arrow).

During surgical exploration, multiple omental adhesions to the abdominal wall and adnexal structures were identified, and adhesiolysis was performed. A systematic exploration of the small bowel was then carried out from the ligament of Treitz to the ileocecal valve, without identifying intraluminal stones or a site of mechanical obstruction. Based on these findings, gallstone ileus was not confirmed intraoperatively as the cause of the obstructive presentation.

During exploration of the appendix, multiple calculous structures were palpated within its lumen. Although the appendix showed no obvious macroscopic inflammatory changes, such as edema, erythema, or purulent material, an appendectomy was performed. Following resection, the appendix was incised to expose its contents, confirming the presence of multiple stones within the lumen (Figure 3, Panel A).

**Figure 3:**
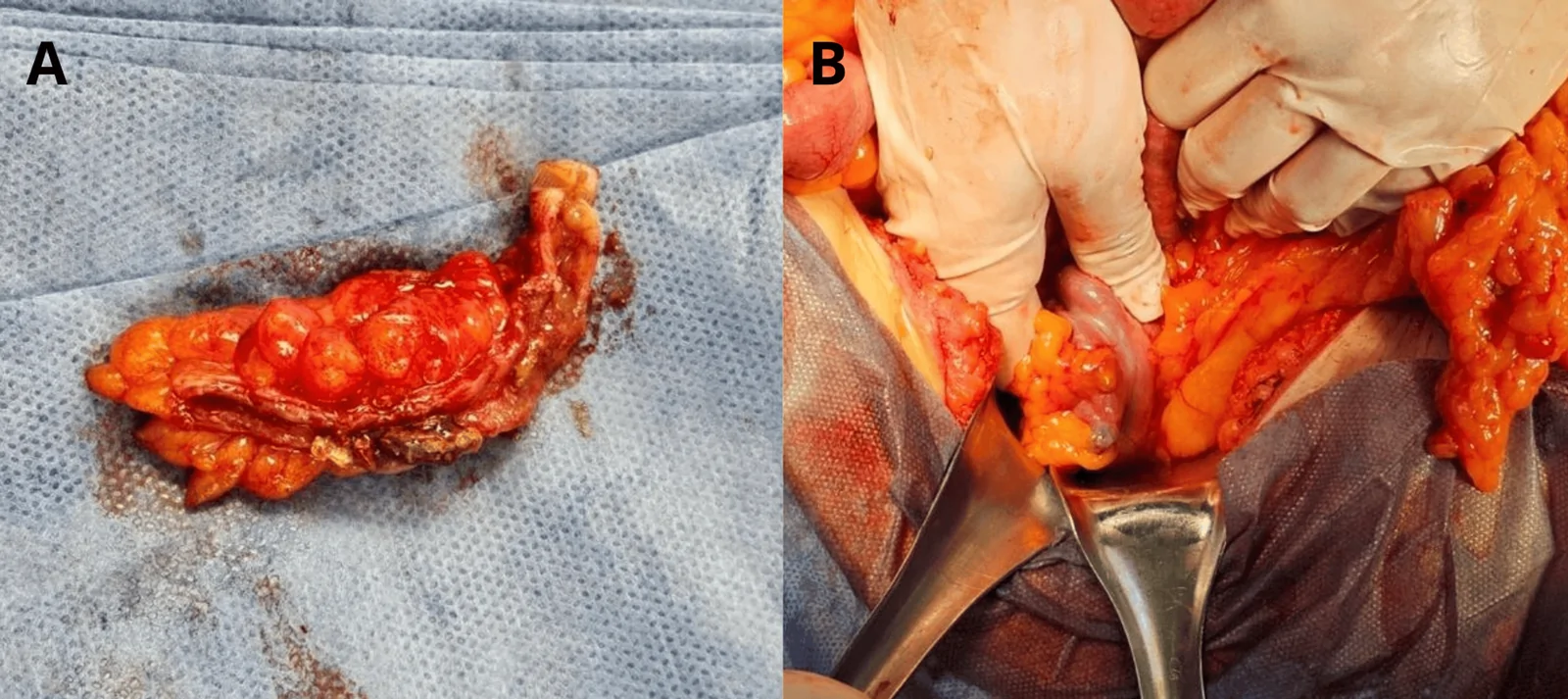
Intraoperative findings (A) Multiple stones within the appendix. (B) Stones lodged within colonic diverticula.

The colon was subsequently explored, revealing multiple calculous structures lodged within colonic diverticula: one in the ascending colon, one in the transverse colon, one in the descending colon, and two in the sigmoid colon (Figure 3, Panel B). The stones were reduced into the intestinal lumen. Taken together with the appendiceal findings, these findings were consistent with multifocal enterolithiasis.

During exploration of the gallbladder, multiple calculi were identified within the gallbladder, including a large calculus impacted at the gallbladder neck. An open cholecystectomy was attempted; however, as the anatomical structures could not be safely identified, the procedure was deferred to a second surgical stage, prioritizing patient safety.

The patient had a favorable postoperative course with adequate clinical recovery and was therefore discharged with outpatient follow-up and subsequent evaluation for definitive management of the gallbladder disease.

At the one-month follow-up visit after hospital discharge, the patient remained asymptomatic, with no reported abdominal pain or other gastrointestinal symptoms. Her postoperative course was uneventful, with good clinical recovery and no complications. Also, the formal radiological report of the computed tomography scan was obtained. The report described cholelithiasis and diverticular disease but did not document the hyperdense intraluminal structures identified during review of the images. This finding was discrepant with the intraoperative findings. Histopathological examination subsequently demonstrated acute fibrinopurulent appendicitis (Figure 4, Panel A) and acute fibrinopurulent periappendicitis (Figure 4, Panel B), confirming acute inflammation despite the absence of evident macroscopic inflammatory changes during surgery.

**Figure 4:**
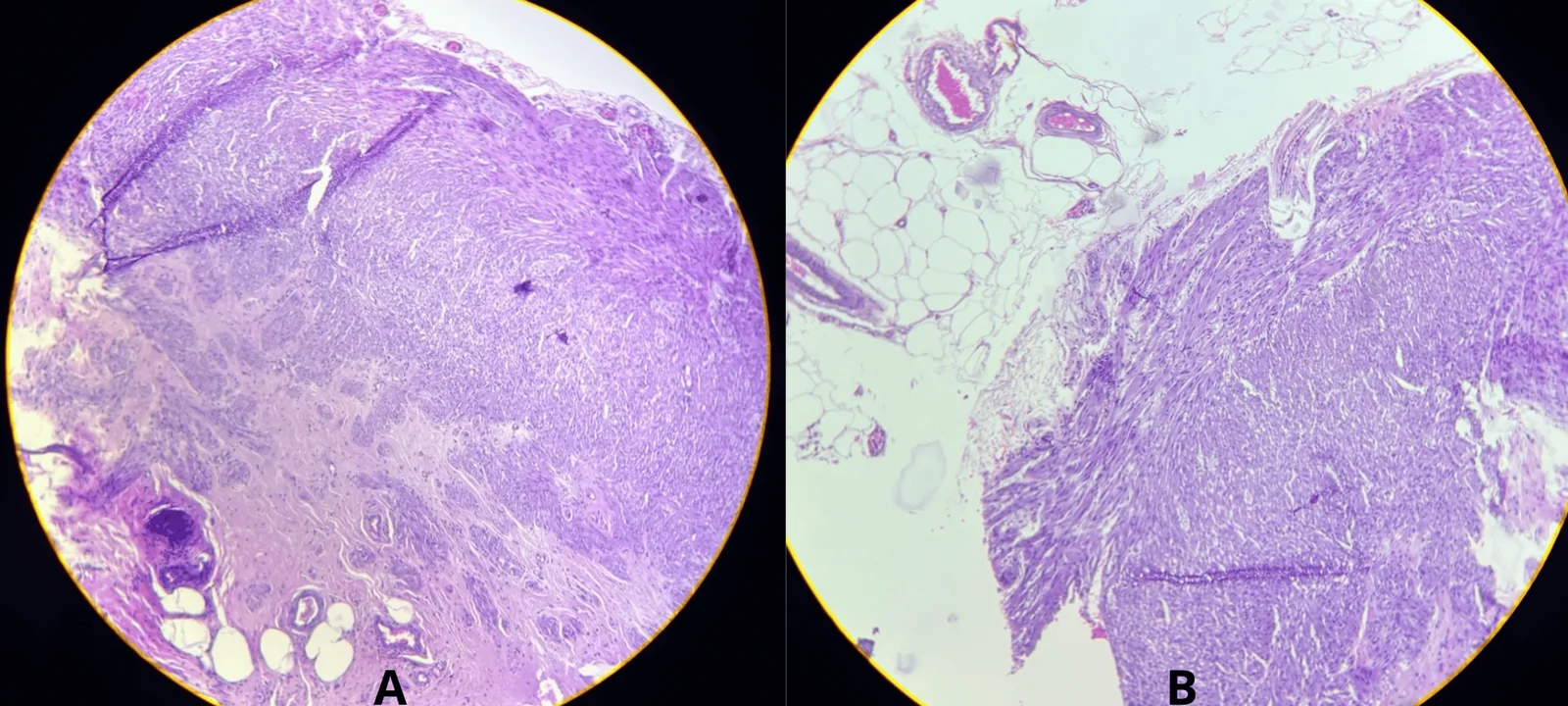
Histopathological examination of the appendix (A) Hematoxylin and eosin (H&E) staining demonstrating acute inflammatory changes involving the appendiceal wall, with prominent inflammatory infiltrates and tissue disruption. (B) Hematoxylin and eosin (H&E) staining demonstrating acute fibrinopurulent periappendicitis, with inflammatory changes extending to the periappendiceal soft tissue.

At the three-month follow-up visit after hospital discharge, the patient reported persistent abdominal pain localized to both iliac fossae, without clinical evidence of an acute abdomen. Given the persistence of symptoms and the history of diverticular disease with multiple intradiverticular stones, colonoscopy was requested as part of the diagnostic workup and remained pending at the time of preparation of this report.

## Discussion

Enterolithiasis is an uncommon condition characterized by the formation of concretions within the gastrointestinal lumen and is generally associated with conditions that promote intestinal stasis, including diverticula, strictures, anatomical abnormalities, blind loops, intestinal anastomoses, and intra-abdominal adhesions. Depending on their location and size, enteroliths may remain asymptomatic or cause abdominal pain, intestinal obstruction, inflammation, perforation, or bleeding [1,2].

The present case is unusual because of the multifocal distribution of gastrointestinal calculi, involving the appendix and multiple colonic diverticula. During surgical exploration, multiple calculi were identified in the appendix, while five additional calculi were found within diverticula located in the ascending, transverse, descending, and sigmoid colon. Enterolith formation within diverticula has been attributed to localized intestinal stasis, which may favor progressive accumulation of intestinal contents and precipitation of mineral components [1,5,6]. Multiple colonic enteroliths have also been described, particularly in the setting of conditions associated with impaired intestinal transit [5]. Although enteroliths may occur as solitary or multiple lesions, the presence of calculi at multiple sites throughout the gastrointestinal tract is uncommon [1,5,6].

An important diagnostic challenge in this case was the coexistence of enterolithiasis and cholelithiasis. Computed tomography demonstrated three hyperdense intraluminal structures within small-bowel loops, together with multiple gallbladder calculi and a large calculus apparently impacted at the gallbladder neck. In the context of abdominal pain, vomiting, obstipation, and absent bowel sounds, these findings raised clinical suspicion for gallstone ileus. However, systematic intraoperative exploration of the small bowel from the ligament of Treitz to the ileocecal valve revealed no obstructing calculus or mechanical transition point. Therefore, gallstone ileus was not confirmed intraoperatively as the cause of the patient's presentation. This distinction is clinically relevant because primary enteroliths form within the gastrointestinal tract as a consequence of local stasis, whereas gallstone ileus results from the passage of a biliary calculus into the gastrointestinal tract, typically through a cholecystoenteric fistula [7,8]. Primary enteroliths may occasionally mimic gallstone ileus clinically and radiologically, particularly when concomitant cholelithiasis is present [3].

The radiological findings in this case also illustrate the diagnostic limitations that may arise when enterolithiasis and cholelithiasis coexist. Enteroliths may have variable radiological appearances depending on their size and composition. Those with a greater mineral content may appear as hyperdense intraluminal structures, whereas enteroliths with a higher proportion of organic material or bile salts may be less conspicuous on computed tomography [1,4]. In our patient, retrospective review of the CT images identified three hyperdense intraluminal structures; however, the formal radiological report described cholelithiasis and diverticular disease without mentioning these structures. Similar diagnostic challenges have been reported in cases of enterolithiasis in which review of cross-sectional imaging was required to recognize the enterolith and establish the diagnosis [9]. The discrepancy between the retrospective image review and the formal report, together with the subsequent intraoperative findings, emphasizes the importance of integrating radiological findings with the clinical presentation and operative examination, particularly when multiple potential sources of gastrointestinal calculi are present [7,9,10].

Another distinctive feature was the presence of multiple calculi within the appendix. Although the appendix did not show evident macroscopic inflammatory changes during surgery, histopathological examination demonstrated acute fibrinopurulent appendicitis and periappendicitis. Appendicoliths have been associated with appendiceal inflammation and may contribute to luminal obstruction [11,12]. Recent reports have also demonstrated that appendicoliths may be associated with significant inflammatory or complicated appendiceal disease [12,13]. In this patient, the coexistence of multiple appendiceal calculi and enteroliths within colonic diverticula raises the possibility that local gastrointestinal stasis contributed to stone formation at multiple sites. However, this interpretation remains speculative because chemical analysis of the calculi was not performed. Therefore, a common composition or shared lithogenic mechanism cannot be established.

The anatomical distribution of the calculi is particularly relevant. Stones were identified within the appendix and diverticula involving four separate colonic segments, while no obstructing calculus was found in the small bowel. This distribution differs from the more typical presentation of symptomatic enterolithiasis, in which a single enterolith or a limited number of concretions may produce intestinal obstruction [1,4-6]. The presence of multiple colonic diverticula may have provided several sites of localized stasis, potentially facilitating the formation and retention of enteroliths at different anatomical locations [1,5,6,14,15]. Similar associations between intestinal diverticula, stasis, and primary enterolith formation have been described in recent case reports [14,15]. Nevertheless, because the exact composition and chronology of formation of the calculi are unknown, this proposed mechanism should be regarded as a possible explanation rather than a definitive pathophysiological conclusion.

The surgical management in this case was guided by the clinical suspicion of intestinal obstruction and the intraoperative findings. Exploratory laparotomy allowed systematic examination of the small bowel and identification of the calculi within the appendix and colonic diverticula. The diverticular calculi were reduced into the intestinal lumen, and appendectomy was performed because of the multiple appendiceal calculi, with acute inflammation subsequently confirmed histopathologically. During exploration of the gallbladder, multiple calculi were identified, including a large calculus impacted at the gallbladder neck. Although an open cholecystectomy was attempted, the inability to safely identify the relevant anatomical structures led to postponement of the procedure. This decision prioritized patient safety and avoided proceeding in the setting of uncertain biliary anatomy and potential bile duct injury.

The patient's persistent abdominal pain during follow-up represents an additional relevant aspect of the case. Although she recovered adequately after surgery and did not develop clinical signs of an acute abdomen, she continued to experience pain in both iliac fossae. Given the extensive diverticular disease and the presence of multiple intradiverticular calculi, colonoscopy was requested as part of the diagnostic evaluation. As this study remained pending at the time of manuscript preparation, it was not possible to determine whether an additional structural abnormality contributed to intestinal stasis or enterolith formation. Further evaluation of the colonic anatomy may therefore provide additional information regarding the underlying mechanism of this unusual multifocal presentation.

Overall, this case highlights the diagnostic complexity of multifocal enterolithiasis in a patient with concomitant cholelithiasis. The presence of intraluminal hyperdense structures on CT, together with gallbladder calculi and a calculus impacted at the gallbladder neck, may strongly suggest gallstone ileus, particularly when diverticular disease is present, and intraoperative exploration does not identify an obstructing gallstone or mechanical transition point. Careful correlation of clinical presentation, radiological findings, and intraoperative anatomy is therefore essential to distinguish primary enterolithiasis from gallstone ileus and to guide appropriate surgical management [1,3,7-10].

Limitations

This report has several limitations. First, chemical analysis of the stones was not performed; therefore, their composition could not be determined, and it was not possible to assess whether the retrieved calculi had characteristics consistent with biliary stones. Second, hepatobiliary ultrasonography was not performed, and there was a discrepancy between the findings identified during review of the computed tomography images and the corresponding formal radiological report. Finally, colonoscopy was requested during follow-up but remained pending at the time of manuscript preparation; therefore, it was not possible to determine whether an additional structural colonic abnormality associated with enterolith formation was present.

## Conclusions

Multifocal enterolithiasis involving the appendix and multiple colonic diverticula is an uncommon finding that may mimic gallstone ileus in patients with concomitant cholelithiasis. In this case, systematic intraoperative exploration demonstrated multiple calculi within the appendix and colonic diverticula, without evidence of an obstructing calculus or mechanical transition point in the small bowel. Histopathological examination confirmed acute fibrinopurulent appendicitis and periappendicitis despite the absence of evident macroscopic inflammation.

This case highlights the importance of considering enterolithiasis in the differential diagnosis of intraluminal calculi, even in the presence of concomitant gallbladder stones. Correlation of imaging findings with intraoperative anatomy is essential for establishing the most likely diagnosis and avoiding premature attribution of intraluminal calculi to biliary stone migration. Although diverticular disease and localized gastrointestinal stasis may have contributed to the multifocal distribution of the calculi, the absence of chemical analysis precludes confirmation of their composition or a shared lithogenic mechanism.
